# No Telescoping Effect with Dual Tendon Vibration

**DOI:** 10.1371/journal.pone.0157351

**Published:** 2016-06-15

**Authors:** Valeria Bellan, Sarah B. Wallwork, Tasha R. Stanton, Carlo Reverberi, Alberto Gallace, G. Lorimer Moseley

**Affiliations:** 1 Sansom Institute for Health Research, University of South Australia, Adelaide 5001, & PainAdelaide, Adelaide, Australia; 2 Department of Psychology, University of Milano-Bicocca, Milan 20126, & NeuroMI—Milan Center for Neuroscience, Milano, Italy; 3 Neuroscience Research Australia, Sydney, Randwick, 2031, Australia; University of Ottawa, CANADA

## Abstract

The tendon vibration illusion has been extensively used to manipulate the perceived position of one’s own body part. However, findings from previous research do not seem conclusive sregarding the perceptual effect of the concurrent stimulation of both agonist and antagonist tendons over one joint. On the basis of recent data, it has been suggested that this paired stimulation generates an inconsistent signal about the limb position, which leads to a perceived shrinkage of the limb. However, this interesting effect has never been replicated. The aim of the present study was to clarify the effect of a simultaneous and equal vibration of the biceps and triceps tendons on the perceived location of the hand. Experiment 1 replicated and extended the previous findings. We compared a dual tendon stimulation condition with single tendon stimulation conditions and with a control condition (no vibration) on both ‘upward-downward’ and ‘towards-away from the elbow’ planes. Our results show a mislocalisation towards the elbow of the position of the vibrated arm during dual vibration, in line with previous results; however, this did not clarify whether the effect was due to arm representation contraction (i.e., a ‘telescoping’ effect). Therefore, in Experiment 2 we investigated explicitly and implicitly the perceived arm length during the same conditions. Our results clearly suggest that in all the vibration conditions there was a mislocalisation of the entire arm (including the elbow), but no evidence of a contraction of the perceived arm length.

## Introduction

Self-localisation–i.e. the perceived location of body parts–acquires proprioceptive information coming from the skin, muscle spindles, muscle contraction, vision, tendons and so on. The perceived orientation of limbs can be modulated by vibrating the tendons that act on the limb (Tendon Vibration Illusion, TVI) [1]. For example at the elbow, vibrating the flexor biceps tendon while keeping the arm still (i.e. strapped in place), induces the perceptual illusion of elbow extension (AVR, Antagonist Vibration Reflex). Conversely, if the extensor triceps tendon is vibrated, the opposite (i.e. illusory elbow flexion) occurs [2]. The TVIs can also induce physiologically impossible perceptions. For example, in the so-called ‘Pinocchio illusion’, vibrating the biceps tendon while holding one’s nose can induce the illusion of one’s nose lengthening as the hand is perceived to be moving away from the head [1]. Similarly, vibrating one’s own biceps tendon while holding a finger of the opposite hand/arm, induces both illusory extension of the vibrated elbow and an impossible stretching of the finger being held [1, 3]. Finally, such illusions penetrate tactile judgements, with tactile distances measured on the finger being perceived as longer during the finger stretching illusion than under control conditions [3].

How then might the brain interpret vibration-induced input arriving simultaneously from the flexors and extensors of the same joint? This question was investigated almost thirty years ago by simultaneous vibration of the biceps and triceps tendons of one elbow [4]. Gilhodes and colleagues reported no illusory movement and no associated muscle activity (i.e. electromyography activity). Later, other studies confirmed this finding, by co-vibrating two antagonist wrist muscles at the same frequency [5, 6]. These studies agreed that, during the vibration of two antagonist muscles, the participants felt as if no movement at all had been performed and, as such, that the position of the vibrated limb was ‘blocked’ in its original position. Fuentes and colleagues [6] interpreted this effect as a failure in updating the position of the limb, due to a position uncertainty lead by two opposing proprioceptive cues.

Interestingly, this same issue was revisited more recently from a different perspective, reporting that, during the dual vibration not only was a positional uncertainty induced, but also that the dual vibration resulted in a ‘telescoping’ illusion of the forearm shrinking towards the elbow [7]. Those authors attributed this altered perception to the inconsistent nature of proprioceptive cues induced by simultaneous vibration of the agonistic tendons and a consequent readjustment of the cortical representation of limb alignment.

**Fig 1:**
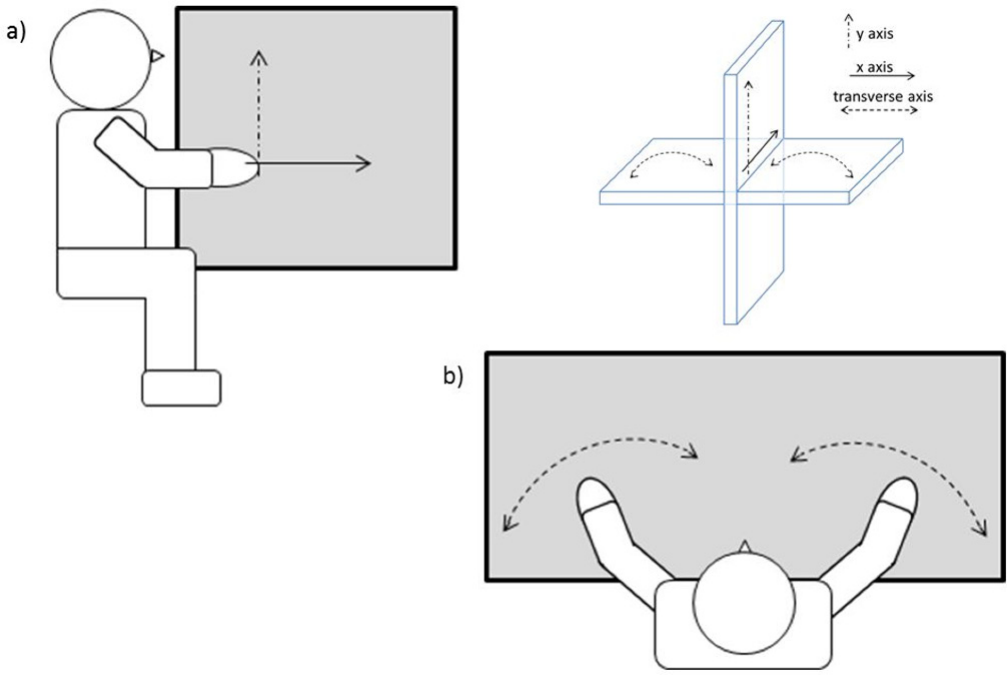
**X (horizontal) and Y (vertical) (a) vs. transverse axes (b).** This figure refers to previous research (i.e. [4,7]).

We see this as a very promising development, not least because of its clear relevance to the perplexing observation of telescoping phantom limbs reported by some amputees [8], a link clearly made by Longo et al. [7]. That the original study [4] evaluated perceived shifts in only the transverse plane however, left open the possibility that the results were confounded by a sideward drift in perceived hand location, a drift that might be unrelated to tendon vibrations [9]. Thus, we sought to interrogate this phenomenon in the sagittal plane by also clarifying aspects not made clear by previous work (see Fig 1). For example, Longo et al. [7] assessed the effects of biceps vibration, dual tendon vibration (i.e. biceps and triceps) and no vibration on the horizontal axis, but did not also assess the effects of triceps vibration. Further, while they assessed the perceived position of the limb on the horizontal axis by means of one task (i.e. a pointing task), they used a different task (i.e. a matching task) on the vertical axis, creating a task confound. These issues are important because it is well known that the triceps vibration leads to the sensation of the arm flexing towards the upper part of the body [10]. By just considering the shift on the horizontal axis (i.e. towards-away from the body), it might be difficult to disentangle whether a shift towards the body is due to the sensation of arm flexing (due to the triceps vibration) or to the actual telescoping effect (due to the dual tendon vibration). In fact, we would expect that the triceps stimulation would induce a shift towards the body and, at the same time, also a shift upwards. On the contrary, taking together the findings of both Longo and colleagues [7], and Gilhodes and colleagues [4], a dual stimulation would induce just a shift on the horizontal axis (i.e. towards the body) due to the telescoping effect. Thus, by not including a triceps vibration condition, only considering results coming from a pointing task and basing conclusions on only the findings on the horizontal axis, we contend that the Longo et al.’s [7] study leaves open the possibility that localisation errors during dual vibration simply reflect a lack of illusory sensation of movement, as declared by Gilhodes et al. [4]. On the grounds that our perceived bodily alignments usually concur with biomechanical constraints [11], we contend that both vertical (Y) and horizontal (X) coordinates would be required to evaluate shifts in perceived location and to differentiate a telescoping limb from illusory joint rotation.

Thus, the aim of the present research was to further investigate this potentially intriguing and clinically relevant discovery of telescoping limbs induced by simultaneous stimulation of antagonist tendons. The first objective was to compare the X and Y coordinates of perceived location of the hand during four different conditions—biceps vibration, triceps vibration, dual vibration and no vibration. We hypothesised that the perceived location of the hand would align with predictions from previous literature–as though the elbow had extended during biceps tendon vibration and as though it had flexed during triceps tendon vibration. This was tested in Experiment 1. Once this was established, the second objective was to evaluate the perceived arm length under these same conditions. Our primary hypothesis, thus, was that dual vibration would induce a shift in perceived location of the hand along the x-axis but not the y-axis–a telescoping effect–as reported by Longo et al. [7]. This was tested in Experiment 2.

## Experiment 1

To verify the presence of a telescoping effect induced by a dual tendon stimulation, we introduced new elements to the original experiment by Longo and colleagues [7]. We used a larger sample including both genders [12], a consistent localisation task for both x and y axis judgements (and for all the conditions), and we measured the localisation judgements during Triceps vibration on both x and y axes (see Table 1).

**Table 1:** Comparison between Longo et al. (2009) and Experiment 1.

	Sample	Task(s)	Axes reported for Biceps vibration	Axes reported for dual vibration	Axes reported for Triceps vibration
	All female	Pointing task (horizontal axis only)	Horizontal axis	Horizontal axis	
*Longo et al*. *(2009)*	n = 8 (exp. 1)				Vertical axis only
	n = 10 (exp. 2)	Matching task	Vertical axis	Vertical axis	
	n = 12 (exp. 3)	(vertical axis only)			
*Experiment 1*	5 females, 8 males	Pointing task	Both, together	Both, together	Both, together
	n = 13				

### Materials and Methods

#### Ethics statement

All the participants gave written informed consent prior to participation. The study was performed in accordance with the ethical standards laid down in the 1991 Declaration of Helsinki and was approved by the institutional Human Research Ethics Committee of University of South Australia.

#### Participants

Thirteen participants (5 female, mean ± SD age = 34 ± 10 years, range 21–51 years) volunteered for this repeated measures randomised experiment. Sample size was determined *a priori* based on previous research [4, 7]. All participants had normal, or corrected to normal, vision and were right handed (self-reported). They had no current or past neurological impairment involving the upper limbs, and no current pain or history of a significant pain disorder. They were naïve about the purpose of the study.

#### Apparatus and experimental setup

**Fig 2:**
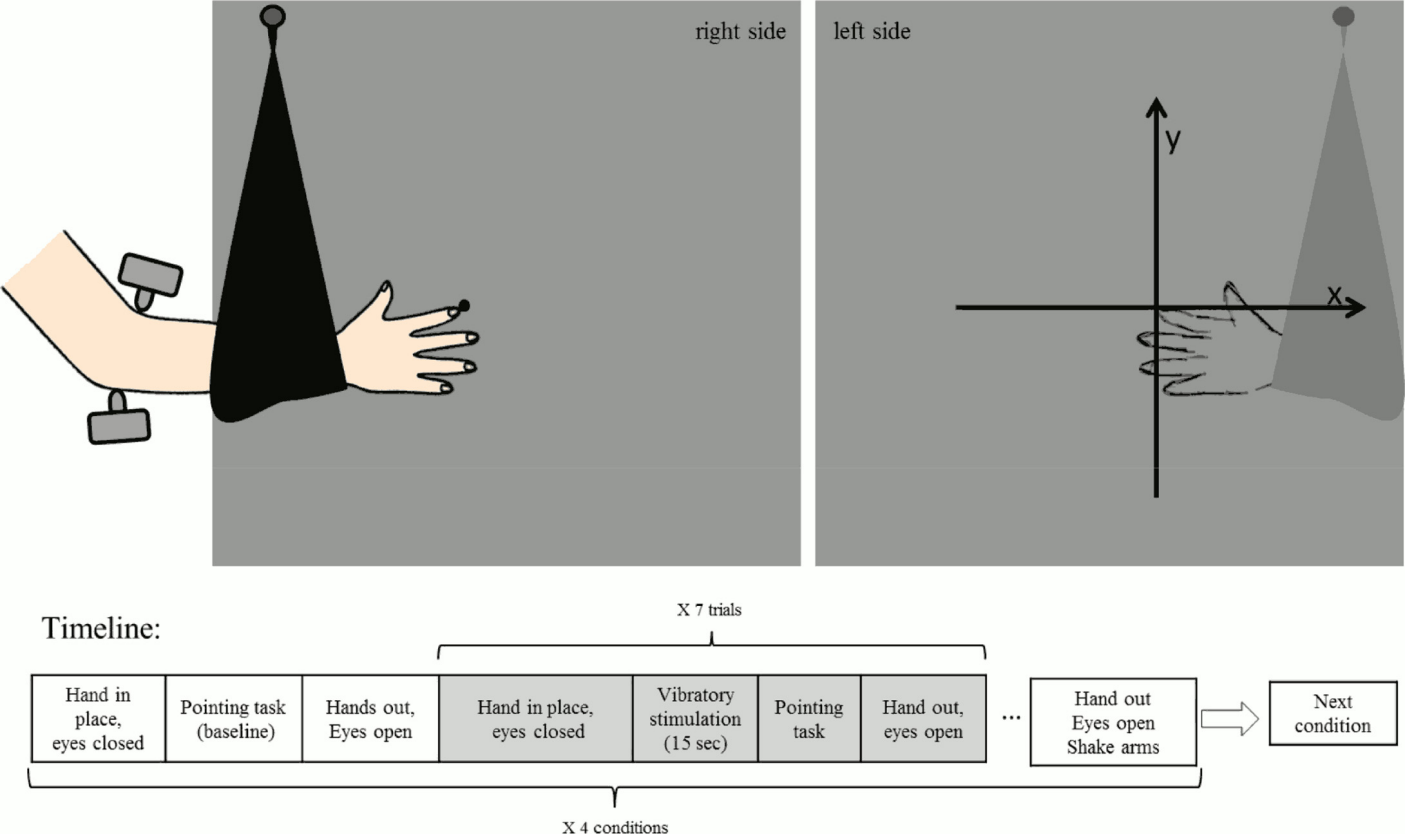
Experiment 1: setup and timeline.

The participants were comfortably seated on a stool, facing the vertical edge of a panel that was aligned with their body midline. They placed their right hand on the right vertical surface of the panel, such that the tip of their right index finger (i.e. the ‘target finger’) made contact with a push pin inserted into the panel about 30 cm away from the edge of the panel. The participant’s right arm and hand were then placed into a looped piece of fabric that was pinned on the panel, such that their elbow formed a ~150^o^ angle (Fig 2). In this way the participant’s right hand and arm were kept in position and slightly supported by the fabric, with the elbow left uncovered. The position of the stool was adjusted for each participant, according to their height and to the length of their arms, in order to standardise these parameters between participants. On the left surface of the panel (Fig 2), two perpendicular 40 cm axes were drawn such that their origin corresponded with the position of the participant’s target finger on the other side of the panel.

#### Tendon vibratory stimulation

The ~40Hz vibration stimuli were delivered over the tendon, between its insertion and the musculotendinous junction, of the prime elbow flexor (biceps brachii ulnar attachment) and the elbow extensor (triceps brachii) of the right arm. Stimuli were delivered by a trained physiotherapist, using commercially available General Purpose Massagers (www.drgraeme.com). The vibration illusion induced with a 40–60 Hz vibration frequency has been recently shown to be more effective in inducing a more vivid, long lasting and with larger perceived illusions of movement than illusions induced at 80–120 Hz [13].

#### Procedure

The participants underwent four different conditions—one control (No vibration) and three experimental (Biceps, Triceps and Dual vibration), in a counterbalanced randomised order. Each condition included an initial baseline measure of the target finger position, followed by seven trials. For each trial a 15 second vibratory stimulation was applied to the triceps, biceps or both tendons simultaneously. At this point participants were instructed to point at the target right index finger with the left index finger (on the left surface of the panel) with their eyes closed (the same procedure was employed to acquire the baseline measure). Importantly, the right index fingertip was considered the origin of the Cartesian system. In order to avoid a sudden disruption of the illusion, the vibration lasted until a second examiner marked the panel on the pointed spot (approximately 18 seconds). After the participants returned their left finger to their left thigh, the experimenter measured the x- and y- axis coordinates of the spot using a transparent plastic ruler (cm). Data were reported on a response sheet. In addition, as a further control, the quadrant in which the localisation occurred was registered by the experimenter. After the measures were recorded, participants were asked to open their eyes, take their arm out of the sling and rest both arms on their lap for a few seconds before the next trial. After completion of 7 trials in a condition, participants were asked to open their eyes and to “shake” both their arms before engaging in the next condition. Importantly, during the No vibration control condition, the participants kept the hand and arm inside the sling and their eyes closed, as they did for the other conditions. The only difference was that no vibration was delivered before the pointing task.

Each pointing response—included the one collected during the baseline -was measured as a shift from the origin (i.e. correct position of the tip of the right index finger) such that, for each localisation, the displacement between the tip of the right index finger and the tip of the left index finger reflected both x and y coordinates. Importantly, positive values indicate ‘closer to the body’ localisations. We conducted two repeated measures ANOVAs that compared the displacement values on the x-axis and on the y-axis across the four conditions (Factor Vibration: Biceps vibration, Triceps vibration, Dual vibration, No vibration).

Additionally, in order to estimate the presence of uncertainty for each condition and for each axis, we considered the standard deviations as a measure of positional uncertainty [6]. Thus, we performed a two (Axis: x-axis, y-axis) by four (Factor Vibration: Biceps vibration, Triceps vibration, Dual vibration, No vibration) repeated-measure ANOVA of standard deviations.

### Results and discussion

**Fig 3:**
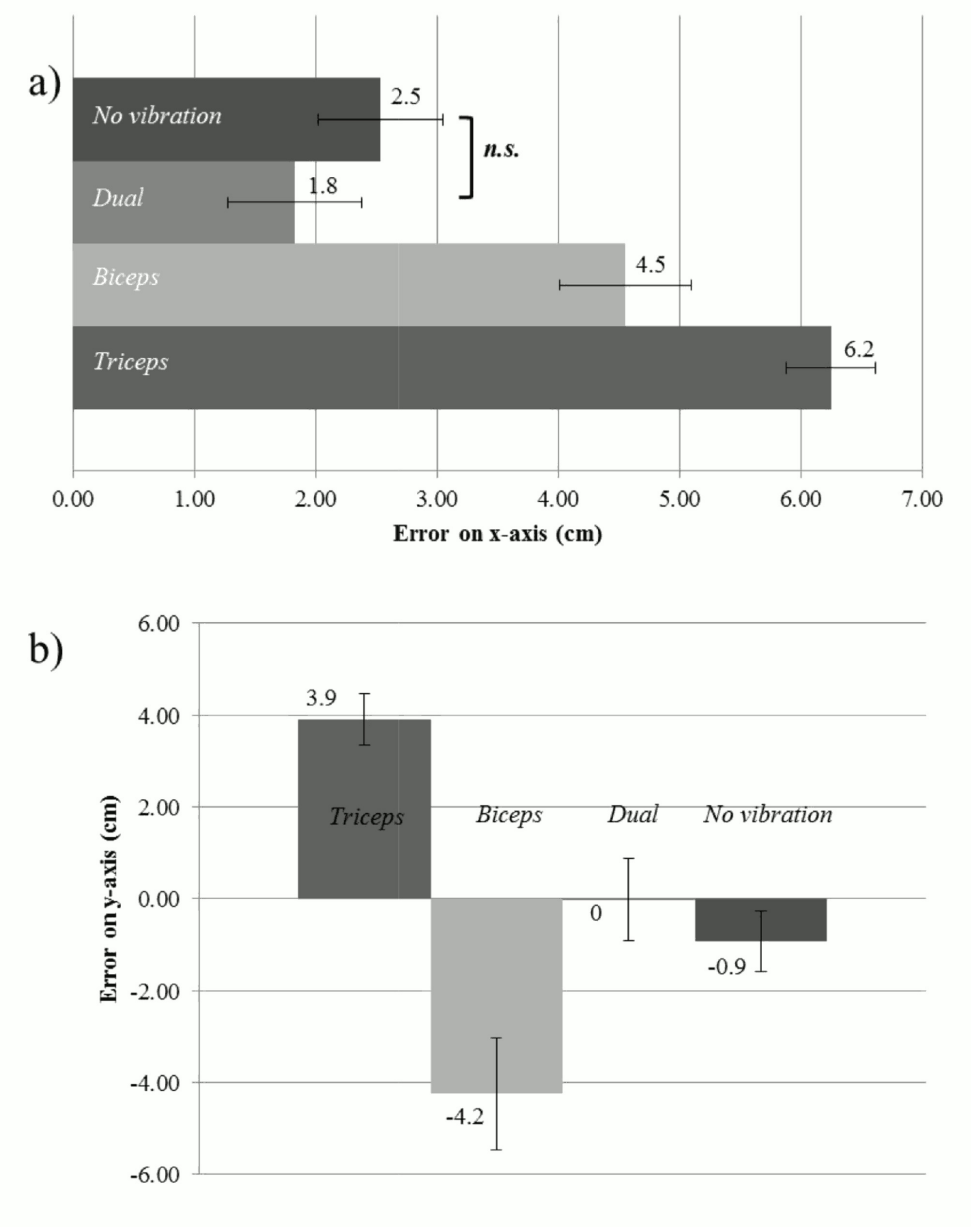
**Effects of vibration on displacement values on x- (a) and y- (b) axis.** ‘0’ represents the position of the target finger on the right surface of the panel. The scores represent the mean error of the pointing task on the X (a) and Y (b) axes and the bars represent the standard error. For each histogram bar the mean is displayed.

x-axis. A significant main effect of Vibration [F(3,36) = 46.9, *p*<0.001, _p_η^2^ = .796] was found. All the displacement values were positive and none of the 95% CIs crossed zero, indicating that they were all significantly different from zero (see Fig 3A), meaning that in all the conditions the target finger’s actual position was significantly mislocalised towards the body. Furthermore, post hoc tests (Bonferroni corrected) revealed significantly larger displacement bias towards the body for the Triceps vibration than for the Dual vibration (*p* = 0.011); than for the Biceps vibration (*p*<0.001); and No vibration (*p*<0.001). In turn, participants showed significantly larger displacement bias for the Dual vibration than for the Biceps vibration (*p*<0.001) and No vibration (*p* = 0.002). No significant difference in the displacement bias towards the body was found between the Biceps vibration and No vibration conditions (*p* = 0.405).

To summarise, the Triceps vibration induced the largest mislocalisation of the target finger towards the body along the x-axis, while a significant shift in the same direction was also induced by the Dual vibration. Conversely, during the Biceps vibration the target finger was not perceived significantly more shifted towards the body than in the No vibration condition.

y-axis Since the assumption of sphericity was violated (Mauchly’s test: χ^2^(5) = 15.27, p = 0.010), a Greenhouse-Geisser correction was applied. A significant effect of Vibration [F(1.732, 20.779) = 3.471, *p*<0.001, _p_η^2^ = .736] was found. The displacement bias for the Triceps vibration and Biceps vibration conditions was significantly different from zero (Fig 2B) (Triceps vibration: M = 3.9 cm, SE = 0.6 cm, 95% CI 2.7 to 7.0; Biceps vibration: M = -4.2 cm; SE = 1.2 cm, 95% CI -6.9 to -1.6 cm), while in both the Dual vibration and No vibration conditions it was not. Furthermore, post hoc tests (Bonferroni correction) showed that the displacement bias in the Triceps vibration condition was larger (i.e. more positive values) than the displacement bias in the Biceps vibration (*p*<0.001), Dual vibration (*p* = 0.002), and No vibration conditions (*p*<0.001). We also found that the displacement bias in the Biceps vibration condition was larger (i.e. more negative values) than the Dual vibration (*p* = 0.001) and the No vibration (*p* = 0.012). The displacement bias in the Dual vibration condition was not significantly different from the displacement bias in the No vibration condition (*p* = 0.152). Thus, a significant upward bias was found for the Triceps vibration condition and a significant downward bias was found for the Biceps vibration condition. To reiterate, both the Dual and No vibration conditions displayed no bias. Finally, in order to compare the amount of displacement bias regardless of its direction (i.e. upward or downward) between the Triceps vibration and Biceps vibration conditions, we transformed the displacement values into absolute values and we then conducted a paired sample t-test on these absolute displacement values (for each condition). There was no difference in the amount of displacement bias between the Triceps vibration (M = 3.9 cm, SD = 2.0) and Biceps vibration (M = 5.3 cm, SD = 2.8) conditions on the y-axis [t(12) = -1.307, *p* = 0.216]. This result clearly shows that the stimulation of the biceps and triceps tendons was not different for the absolute amount of displacement bias, but only for the direction of the error (i.e. upward for the triceps and downward for the biceps).

**Fig 4:**
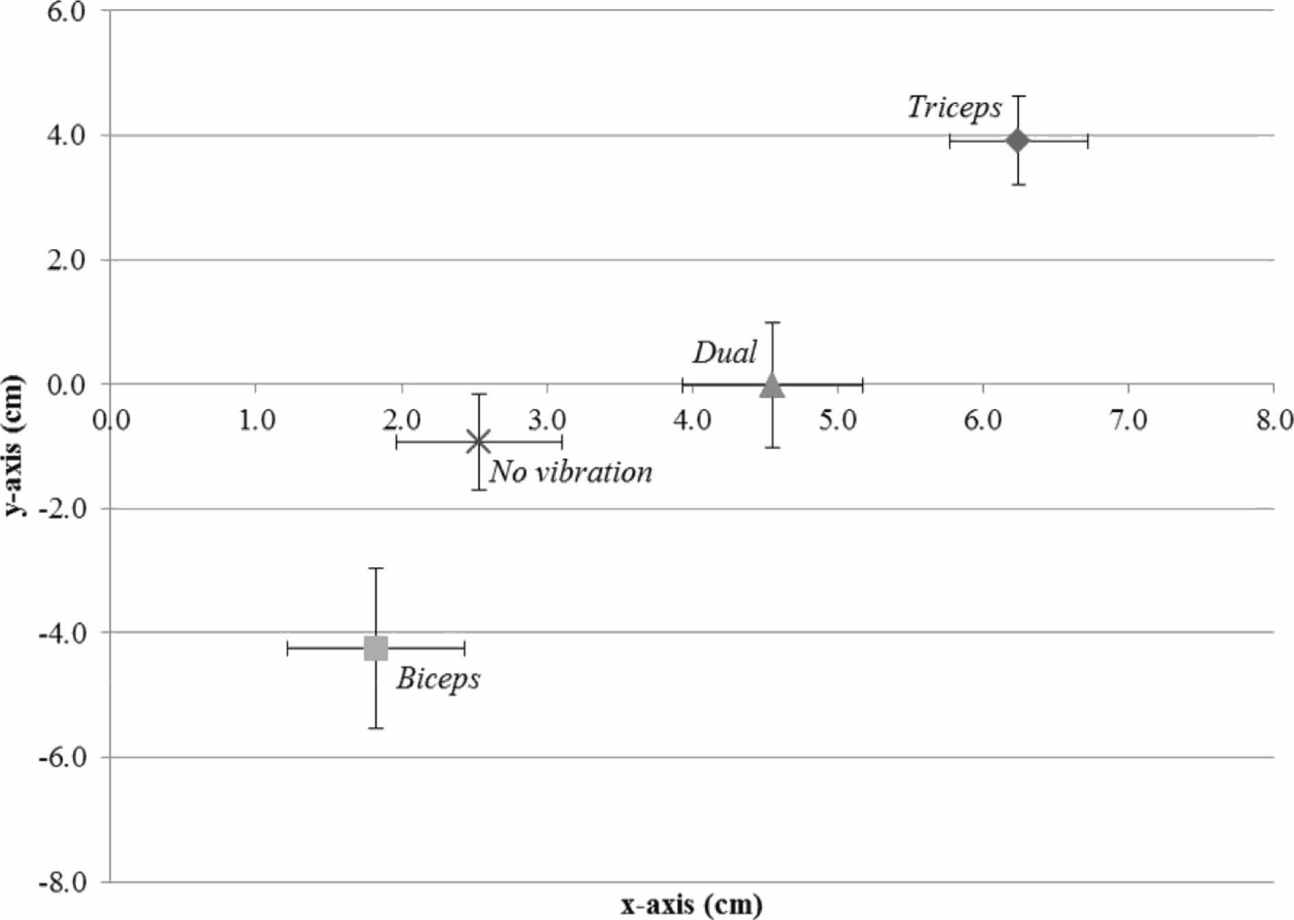
Errors measured on the x- (positive values indicate ‘closer to the body’ localisations) and y-axis plotted together on Cartesian system.

Our data show that the single vibration of the tendons induces equal and opposite mislocalisations of the target finger. Triceps tendon induces a large upward shift (i.e. illusory flexion) while the biceps tendon a downward shift (i.e. illusory extension). Furthermore the downward displacement bias reported for the Dual vibration condition was not significantly different than the one reported for the No vibration condition. This supports previous findings in which muscle activity was not reported during paired vibratory stimulation, but it was reported during single tendon stimulation [4], leading to the idea that double vibration is able to suppress the kinaesthetic illusion. Thus, interestingly, in the Dual condition the participants seemed to correctly perceive their arm position on the y-axis, while they perceived it as shifted towards the body on the x-axis. This finding would be in line with Longo et al. [7], about the presence of a telescoping effect (e.g. a displacement of the arm) in case of contrasting positional cues. The pattern of results across conditions is presented graphically in Fig 4: the triceps vibration induces the sensation of elbow flexion, leading the index finger to be perceived upward and closer to upper part of the body.

Finally, the ANOVA performed on the standard deviations revealed main effects of Axis [F(1,12) = 33.8, *p* < .001, _p_η^2^ = .738] and Vibration [F(3,36) = 3.48, *p* = .026, _p_η^2^ = .225] and a significant interaction between the two factors [F(3,36) = 5.27, *p* = .004, _p_η^2^ = .305].The standard deviations were larger for the y-axis (mean = 1.0 cm, SE = 0.1 cm, 95% CI 0.7 to 1.3 cm) than for the x-axis (mean = 1.6 cm, SE = 0.1 cm, 95% CI 1.3 to 1.9 cm). A planned pairwise comparison performed on the Vibration factor, revealed that the standard deviations were larger only during the Biceps vibration than when no vibration was applied at all (i.e. No vibration) (*p* = 0.006). This would suggest a higher variability across trials in the Biceps vibration condition than in the No vibration condition. Post-hoc t-tests showed that in all conditions, except the No vibration condition (*p* = 0.27), the standard deviations were larger for the y-axis than for the x-axis (Triceps: *p* = 0.032, Biceps: *p* < 0.001, Dual: *p* = 0.012). This last result confirms what was already shown by the main effect of Axis. Therefore, the positional uncertainty seems to be higher on the y-axis than on the x-axis, but generally comparable between different conditions.

Taken together, data from Experiment 1 confirms that an illusion of displacement takes place when a single tendon or antagonist tendons are vibrated. However, while Experiment 1 replicated previous results by Longo et al. [7], it is unclear whether the participants experience “true” telescoping (in that representation of the forearm contracts) or whether they perceive that their *entire* arm is shifted towards the body. To shed light on this issue, we ran a second experiment.

## Experiment 2

In Experiment 2, we evaluated the same conditions as in Experiment 1, but we had participants point to the perceived location of their fingertip, their wrist and, their elbow. In this way, we were able to compute the relative distances between the three points and, thus, compare the perceived size of each segment (i.e. from fingertip to wrist and from wrist to elbow). In addition, participants performed a visual task in which they had to identify the perceived length of their forearm. Finally, participants’ self-reported appraisals of the experiment were collected.

### Materials and Methods

#### Ethics statement

All participants gave written informed consent prior to participation. The study was performed in accordance with the ethical standards laid down in the 1991 Declaration of Helsinki and was approved by the institutional Human Research Ethics Committee.

#### Participants

Fifteen new participants (8 female, mean ± SD age = 21 ± 2, range 19–25) volunteered for this repeated measures randomised experiment. Sample size was calculated *a priori*, on the basis of Experiment 1, to obtain a statistical power of at least 95% for medium effect size (f = 0.25). Inclusion criteria were the identical to Experiment 1 and participants were naïve about the purpose of the study.

#### Apparatus and experimental setup (Fig 5)

Participants were seated on a chair, facing the vertical edge of a transparent panel that was aligned with their body midline. Their right upper arm was placed on a soft adjustable support, and the elbow, forearm and hand were placed on the right surface of the panel. When a comfortable position was obtained, one experimenter marked the position of the participants’ right index fingertip, middle of right wrist and right elbow (at the level of the medial epicondyle bone) (targets) on the right side of the panel with a non-permanent whiteboard marker. As additional reference points, the participant’s arm was also outlined with the marker on the transparent panel around the finger, wrist and elbow. Participants were blindfolded and the first experimental block started.

**Fig 5:**
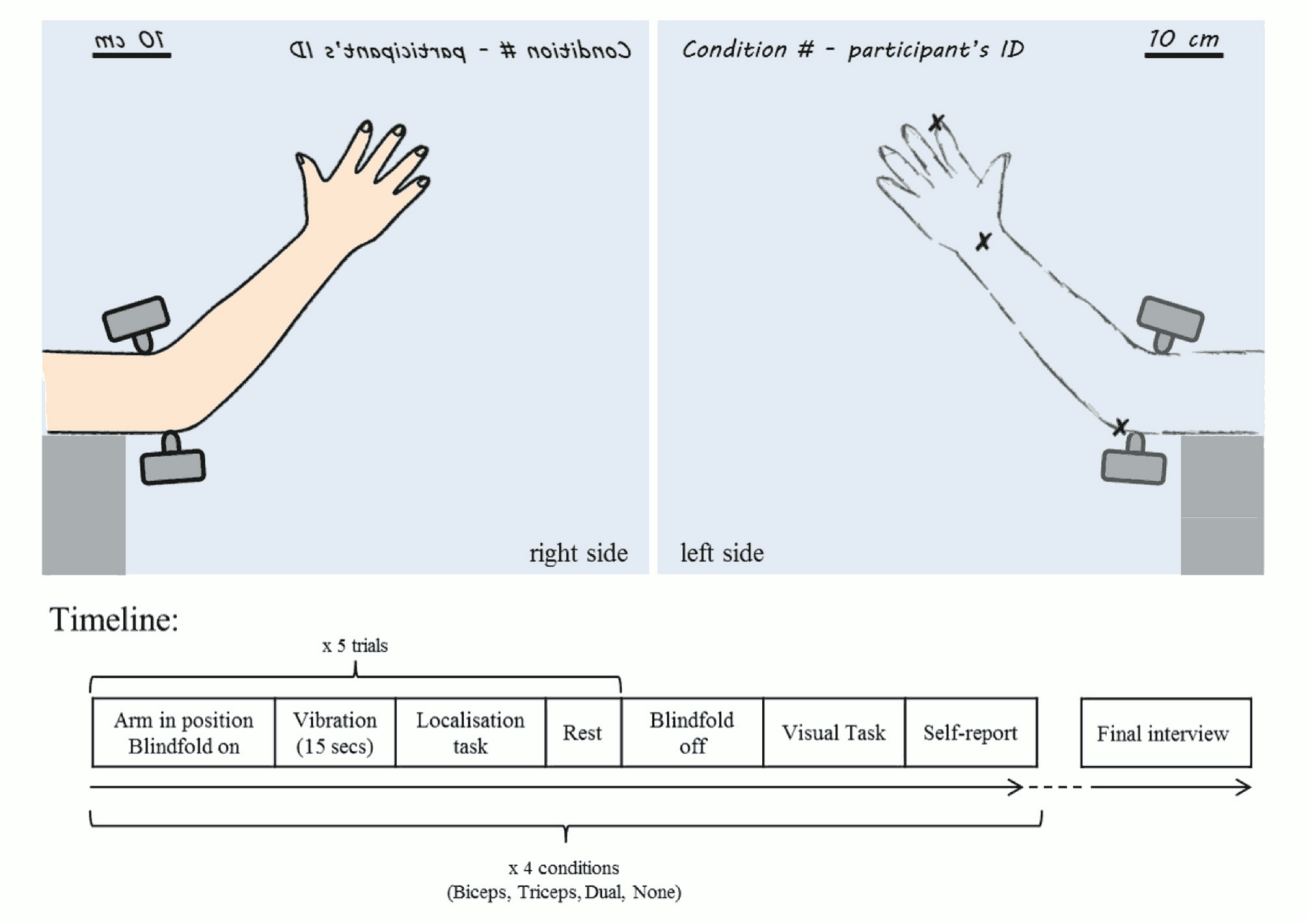
Experiment 2: setup and timeline.

The tendon stimulation was as described in Experiment 1 and was performed by the same experimenter, but lasted ~24 seconds (i.e. 6 seconds more than in Experiment 1, to allow for sufficient time for participants to point to the wrist, elbow, and fingertip).

#### Localisation task

The localisation task was identical to that described in Experiment 1, except that participants, after 15 seconds of vibration, were asked to point at three targets (fingertip, wrist and elbow). Each participant completed a total of four randomised conditions (same as in Experiment 1). In contrast with Experiment 1, for each block, five localisation judgements were made for each target. Pilot data did not show any significant effect on average localisation errors yielded by this modification.

After the participants pointed at each spot of interest, a second experimenter (same of Experiment 1) marked on the panel each localisation labelling it with the trial number. After each stimulation, participants rested for 15 seconds, but were required to keep their right arm and the blindfold in place. The experimenters constantly checked that the arm and hand were kept in the correct position. At the end of each block, participants were directed away from the testing area so that their localisation responses were shielded from view. In this way, the participants were unaware of their own localisation judgements.

#### Visual scale task

**Fig 6:**
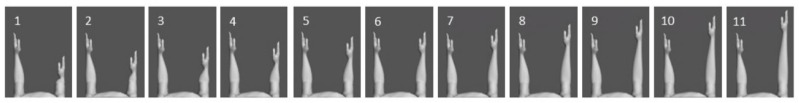
Visual scale.

Participants were presented with a sequence of 11 pictures that depicted two bent arms from a first person perspective (see Fig 6). The median picture (n. 6) showed two identical right and left forearms from first person perspective. Pictures were created with the application for Android Pose Tool 3D (retrieved May 5^th^, 2015 from https://play.google.com/store/apps/details?id=com.alienthink.posetool3d&hl=it) and the length of the right arm was then manipulated using the software Gimp 2.8.4 (retrieved May 29^th^ 2013 from http://www.gimp.org/). Starting from picture n. 6, the pictures on the left half of the scale showed an increasingly shorter right forearm (with a 1/5 decrement from picture n. 5 to n.1), while the pictures on the right half of the scale showed an increasingly longer right forearm (1/5 increment from picture n. 7 to 11). The visual scale was attached on a wall with the pictures in ascending order from left to right–starting from the one with the shortest forearm to the one with the longest forearm. Participants chose the picture that best represented how their right arm felt during the vibratory stimulation for that block; an experimenter recorded the response.

#### Self-report

In order to avoid any bias from the experimenter, the participants were asked to write down a brief self-report regarding their sensations on the vibrated arm for that block, as a memory aid. It was emphasised that the intent of the task was to capture sensations experienced so that could be discussed later. The experimenter, then, asked the participants to verbalise their written report and took note of it. The slip of paper was then hidden from the participants’ view.

At the end of the experimental session, participants were given back their own slips of paper containing their descriptions about each condition and they were again asked to talk about the sensations they felt during the experiment, specifically comparing between different conditions. Participants were asked to focus on two points: (1) whether in any of the conditions they felt that the vibrated arm was moving and, if so, in which one(s); (2) whether in any of the conditions they felt a change in shape of the vibrated arm and, if so, in which one(s). An experimenter just took note of the participant’s answers.

#### Data handling

Localisation task. Data from the localisation task were used to compare the amount of localisation error for the fingertip, wrist and elbow on both the x and y-axis and to indirectly compute the perceived implicit distances of the hand (i.e. from fingertip to wrist) and of the forearm (i.e. from wrist to elbow). In the four photos taken for each participant (one for each condition), the true positions of the three targets (fingertip, wrist and elbow) were marked and each represented the origins of a different Cartesian system. For each target, the experimenter (blinded to the condition) measured the distance from the origin of each of the five localisations made by the participant on both the x- and y-axis. This resulted in five measurements on the x-axis and y-axis for each of the four conditions. In order to have comparable distances, each measurement was then scaled according to the 10 cm segment marked on the photographed panel. The average localisation error on both axes was then calculated for each condition. At the end of this process, the experimenter matched the data with the condition.

Two repeated measure ANOVAs (i.e. one for the x- and one for the y-axis) were performed for each target in order to compare the average localisation error between the four conditions.

To evaluate the perceived length of the vibrated arm, the experimenter–blinded to the condition–measured the distance between the fingertip, wrist and elbow localisations within the same trial. That is, for each condition, five (i.e. one per trial) implicit measures of the perceived hand and forearm lengths were obtained and the averages were then calculated. Percentage differences ([real length–perceived length] / real length) of hand and forearm length were then computed. Finally, the experimenter matched the data with the condition.

Two repeated measures ANOVAs (one for the hand and one for the forearm) were performed in order to compare differences in the perceived lengths between the four conditions (i.e., Triceps, Biceps, Dual and No vibration).

Visual scale task. For each participant we obtained four ratings (one per condition). In order to detect a difference between the baseline visual representation of the participants’ arm and possible changes due to the vibratory stimulations, we subtracted the number obtained after each condition from the number obtained after the No vibration condition. Positive digits indicated an increase in the size of the visual representation of the arm, while negative digits a decrease in the visual representation of the arm. We then divided the results into category accordingly (‘longer arm’, ‘shorter arm’ and ‘same length arm’). For each vibration condition (Biceps, Triceps and Dual) we performed a chi square test, to investigate whether the distribution of each category was significantly different from the distribution of the other two categories.

Self-report. The answers obtained by self-report are presented in S1 Appendix. The answers for the question regarding changes in shape and /or length have been compared with the results from the localisation task and from the visual task.

### Results and discussion

Overall, the results depict a consistent picture that the limb does not telescope during vibration of both tendons. That is, the localisation of the fingertip, wrist and elbow, taken together, show that the entire limb is, in fact, mislocalised in all the vibration conditions. In addition, the perceived length of the limb was not shorter during vibration of both tendons, than it was during vibration of just one tendon. In agreement with this, the ratings of the visual scale and the self-reports did not reveal any telescoping effect.

#### Fingertip localisation (Fig 7A)

Vibrating the Triceps tendon induced the largest mislocalisation of the fingertip *towards* participants’ body. Vibration of the Biceps tendon induced the largest mislocalisation of the fingertip *away* from the body (i.e. in the opposite direction). On the other hand, vibrating the biceps tendon induced the largest downward mislocalisation, while vibrating the triceps tendon or no vibration at all produced the most upward mislocalisation. In all conditions, however, the finger was mislocalised, being perceived at a lower level than where it actually was.

**Fig 7:**
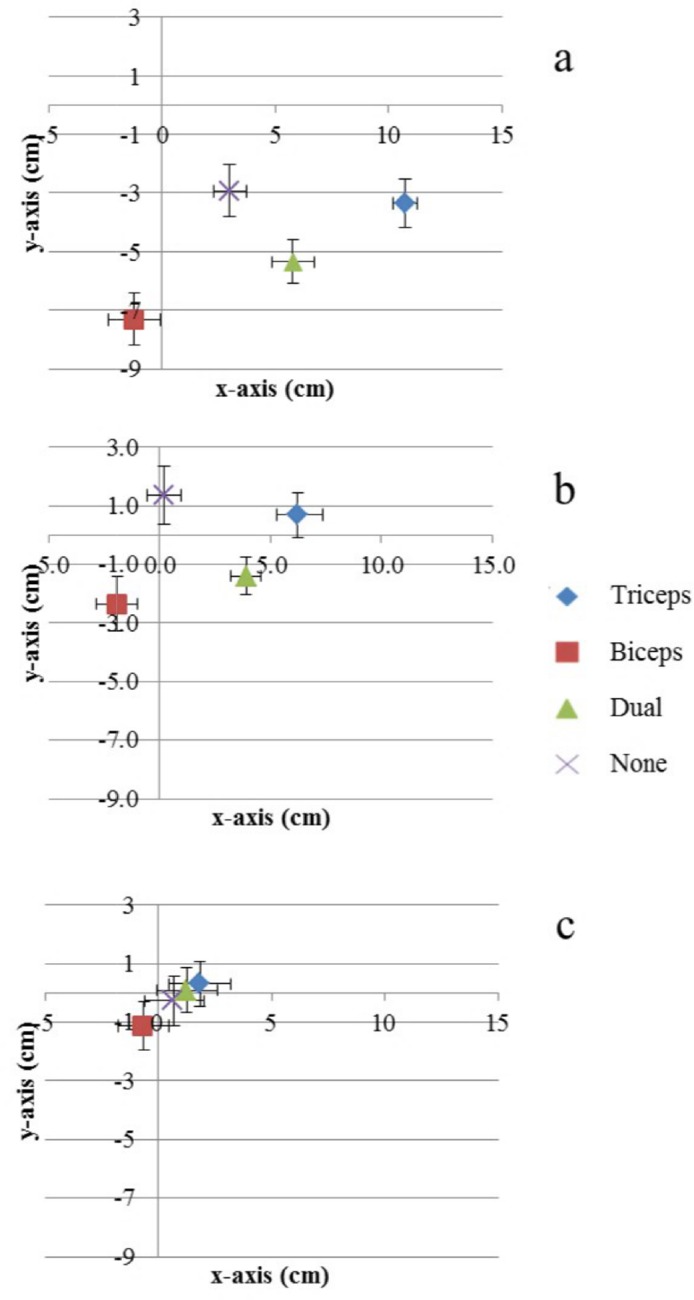
**Errors measured on the X and Y axis for fingertip (a), wrist (b) and elbow (c) localisations.** Bars indicate standard error.

x-axis**.** The assumption of sphericity was violated (Mauchly’s test: χ^2^(5) = 12.26, p = 0.032), a Greenhouse-Geisser correction was applied to repeated measure ANOVA analyses. Mean localisation errors on x-axis differed significantly between conditions [F(1.839, 25.749) = 53.362, p<0.001, _p_η^2^ = 0.904]. Post hoc tests (Bonferroni corrected) revealed that vibration applied on the Triceps tendon elicited larger positive localisation error than vibration on the Biceps tendon (p<0.001), Dual vibration (p<0.001) or No vibration (p<0.001). The vibration on the Biceps tendon, in turn, elicited the largest negative localisation error (D: p<0.001, N: p = 0.001). The positive localisation error on the x-axis was larger in the Dual vibration condition than it was in the No vibration condition (p<0.001).

y-axis**.** The assumption of sphericity was again violated (Mauchly’s test: χ^2^(5) = 11.98, p = 0.036) and a Greenhouse-Geisser correction was applied to repeated measure ANOVA analyses. Mean localisation errors on y-axis differed significantly between conditions [F(2.012, 28.168) = 10.395, p<0.001, _p_η^2^ = 0.426]. Post hoc tests (Bonferroni corrected) revealed that vibration applied on the biceps tendon elicited the largest negative localisation error on the y axis (Triceps, p = 0.004; Dual, p = 0.015; No vibration, p = 0.001). In the Dual condition, the localisation error was significantly more negative than in the Triceps condition (p = 0.013) or the No vibration condition (p = 0.013). No significant difference was detected in the amount of localisation error on the y-axis between Triceps (M = -3.4 cm, SE = 0.9 cm, 95% CI: -3.4 to 0.9 cm) and No vibration (M = -2.9 cm, SE = 1.0 cm, 95% CI: -4.9 to -1.0 cm) conditions and both were significantly different from zero.

The data suggest a mislocalisation upwards and towards the body during triceps vibration and a mislocalisation downwards and away from the body during biceps vibration. When both triceps and biceps tendons were vibrated at the same time, the fingertip was localised in a median position between the single tendons localisations. The control condition (no vibration) was the most accurate (smallest localisation error) on the y-axis and, even though there was a significant mislocalisation towards the body (x-axis), the localisation error was not significantly different from zero.

#### Wrist localisation (Fig 7B)

As for the finger localisation, during the Triceps vibration condition, the wrist was mislocalised towards the body, while during the Biceps vibration condition it was mislocalised in the opposite direction. Again during the Dual condition the wrist was localised between the Triceps and the Biceps localisations. The wrist had greater mislocalisation during the Triceps vibration condition (upward and towards the body) than any other condition. The wrist was also mislocalised (downward and away from the body) during the Biceps vibration condition than any other condition. In the Dual condition the wrist was localised towards and downwards.

x-axis. Since the assumption of sphericity was again violated (Mauchly’s test: χ^2^(5) = 14.19, p = 0.015), a Greenhouse correction was applied to repeated measure ANOVA analyses. Mean localisation errors on x-axis differed between conditions [F(1.751, 24.513) = 26.403, p<0.001, _p_η^2^ = 0.653]. Post hoc tests (Bonferroni corrected) revealed that vibration applied on the triceps tendon elicited larger positive localisation error than vibration on the biceps tendon(p<0.001), dual vibration (p = 0.033) and no vibration(p<0.001). The Biceps condition, conversely, caused the largest localisation error on the opposite direction (i.e. away from the body) (Dual, p<0.001; No vibration, p = 0.031). The average localisation in the Dual condition was significantly more shifted towards the body (i.e. more positive) than in the No vibration condition, in which the localisation error was not significantly different from zero (M = 0.2 cm, SE = 0.8 cm, p<0.001, 95% CI: -1.5 to 1.9 cm).

y-axis. Sphericity was assumed (Mauchly’s test: χ^2^(5) = 10.82, p = .056). Mean localisation errors on y-axis differed significantly between conditions [F(3,42) = 6.884, p = 0.001, _p_η^2^ = 0.330]. Post hoc tests (Bonferroni corrected) revealed that vibration applied on the biceps tendon caused a significative larger negative localisation error than triceps vibration (p = 0.022) and than no vibration (p = 0.008). However, there was no significant difference between the localisation error obtained in the Biceps and Dual conditions or between the Triceps and No vibration conditions. On the contrary, the average localisation error in the Dual condition was significantly different (i.e. more negative) than the average localisation errors in both the Triceps (p = 0.004) and No vibration (p = 0.006) conditions. Except the Biceps vibration conditions (M = -2.3 cm, SE = 0.9 cm, 95% CI: -4.4 to -0.3 cm), in the other three conditions the average localisation error was not significantly different from zero.

#### Elbow localisation (Fig 7C)

x-axis. Sphericity was assumed (Mauchly’s test: χ^2^(5) = 3.74, p = .589). Mean localisation errors on x-axis differed between conditions [F(3,42) = 5.861, p = 0.002, _p_η^2^ = 0.295]. Post hoc tests (Bonferroni corrected) revealed that vibration applied on the triceps tendon induced more positive localisation error (i.e. localisation towards the body) than biceps vibration (p = .022,) and no vibration (p = 0.042), but it was not different from the dual vibration. The localisation error during biceps vibration was smaller (farther from the body) than during dual vibration (p = 0.017) but not significantly different than during no vibration. Finally, the localisation errors during the Dual and No vibration conditions were not significantly different. Importantly, the mean localisation error was not significantly different from zero during all conditions.

y-axis**.** Mean localisation errors on y-axis did not significantly differ between conditions (p>0.05).

#### Perceived arm length

While the perceived length of the hand (segment from fingertip to wrist) did not change between conditions, the perceived length of the forearm (segment from wrist to elbow) did. In particular, a shrinking in the perceived length of the forearm occurred during the Dual condition, but this same shrinking was also present in the Triceps, Biceps and No vibration conditions.

Fingertip to wrist (hand)**.** Sphericity was assumed (Mauchly’s test: χ^2^(5) = 7.699, p = 0.175). Mean percentage difference was not significantly different between conditions [F(3,42) = 2.495, p = p = 0.073].

**Fig 8:**
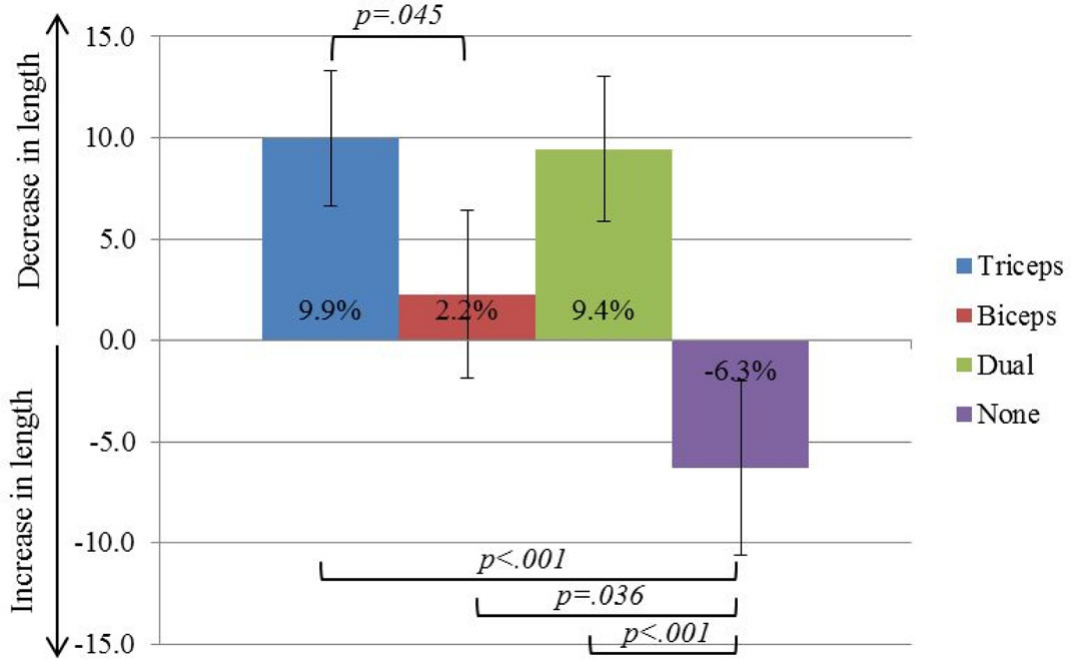
Changes in perceived forearm length (fingertip to wrist). Bars indicate standard error.

Wrist to elbow (forearm) (Fig 8). Sphericity was assumed (Mauchly’s test: χ^2^(5) = 1.741, p = 0.844). Mean percentage differences in length of the forearm significantly differed between conditions (F[3,42] = 10.929, p = 0.001, _p_η^2^ = 0.438). Post hoc tests (Bonferroni corrected) revealed that the perceived length of the forearm in the Triceps condition did not differ from the perceived length in the Dual condition (p = 0.850), while in the Dual condition the forearm was perceived to be smaller than in the No vibration condition (p<0.001) but no significant difference was detected between the Dual and Biceps conditions. Vibration applied on the triceps tendon lead to a perceived smaller forearm than biceps vibration (p = 0.045), and no vibration (p<0.001). The perceived length of the forearm in the Biceps condition was smaller than in the No vibration condition (p = 0.036). Perceived length both from Biceps and No vibration conditions did not significantly differ from zero.

#### Visual task

Based on our data, participants’ visual representation of their vibrated arm was not correlated with the site that was vibrated. A Pearson’s R test was performed to calculate whether there was an association between the perceived arm length, as judged by the visual task and the vibration site, than when no vibration was applied. No difference was found between the frequencies of responses between conditions [χ^2^ (4) = 4.181, p = 0.382].

#### Self-report

Participants’ responses to the questions that were asked at the end of each experimental session are found in S1 Appendix, Table A.

Despite clear localisation errors (see above), most participants did not experience a conscious illusory arm movement. This could be explained by the low frequency stimulation (<100Hz)[14] and it is consistent with the literature in which several parameters have been reported to affect the illusion [13, 15, 16].

Participants were inconsistent with their responses regarding perceived shape and length of their vibrated arm (see Table B in S1 Appendix). During triceps vibration 26.6% of participants’ self-report were consistent with the hand perceived length, 13.3% with the forearm perceived length and 46.6% with the ratings at the visual scale. For the Biceps vibration, the 6.6% of the participants’ self-report were consistent with the hand perceived length, 33.3% with the forearm perceived length and 40% with the ratings at the visual scale. Finally, for the Dual vibration, the 26.6% of the participants’ self-report were with the hand perceived length, 20% with the forearm perceived length and 13% with the ratings at the visual scale.

This data indicates that, during the triceps and biceps vibration, the two explicit measures of the perceived arm length (i.e. responses at the visual scale and self-report) were more consistent, even though less than half of the participants were actually consistent. In contrast, during the Dual vibration condition, participants were inconsistent with both their explicit (responses at the visual scale and self-report) and implicit (calculated perceived length) responses.

## General Discussion

We aimed to investigate and characterise the important discovery of telescoping limbs induced by simultaneous stimulation of antagonist tendons [7]. Specifically, we wanted to (1) replicate and extend the results found by Longo and colleagues [7], and (2) investigate perceptions of arm length by mean of different methods. Our results show that participants had an altered sense of limb position during tendon vibration, but perceived arm length remained unaffected. That is, there was no telescoping of the vibrated limb.

Longo et al. [7] demonstrated that, under positional uncertainty induced by tendon vibration, the felt dimensions of a body part are affected in a particular way–a shortening of the felt length of the arm as result of a dual tendon vibration. This interesting effect, indeed, has not been reported elsewhere. The results from Experiment 1 of this study confirm and extend some of the effects reported by Longo and colleagues [7]. The illusion of arm flexion and extension consequent to single tendon vibration was replicated. Still, even if the results obtained during dual vibration raised the possibility of a telescoping effect, an illusory backward extension of the arm and elbow could not be excluded. In Experiment 2, by considering perceived arm localisation not only at the fingertip, but also at the wrist and elbow, we gained an understanding of what was happening to the whole arm during vibration, rather than what was happening to just the fingertip. Interestingly enough, the combined results showed that the whole arm felt translated during the vibration conditions. As clearly shown in Fig 7, all the conditions seemed to follow the same mislocalisation trend for all targets (i.e. fingertips, wrist and elbow). This means that even the elbow was felt to be mislocalised, similarly to the fingertip and wrist. Thus, not only the arm but also the shoulder seems to be affected by the tendon vibration and the shoulder itself may have produced the perceived translation of the whole arm. This may be due to the particular length-tension relationships of each muscle at the arm angles we tested in (the arm was always flexed), or perhaps different local tissue issues (Biceps tendon is more superficial than Triceps tendon, for example). However, further studies will be needed in order to explore that possibility. One interesting aspect is that these muscles (i.e. Biceps and Triceps) cross more than one joint. Remarkably, we could see no evidence in the literature that, when tendons of multi-joint muscles are vibrated, consideration is given to the proprioceptive impact on more than one joint.

The distances between the perceived position of fingertip and wrist, and wrist and elbow were measured such that *implicit* measures of the perceived length of the hand (fingertip to wrist) and of the forearm (wrist to elbow) were obtained. As one would expect, the data showed that the hand length was not perceived differently between the vibration and no vibration conditions. This was quite predictable—the hand tendons were not vibrated. However, even if during the dual tendon vibration, the forearm was indeed perceived as being shorter than usual, this same effect was also present with both triceps and biceps single tendon vibrations. This implicit measure of perceived limb length strongly denies the idea of a contraction of arm representation due to the vibration of antagonist tendons. However, it is in line with the idea of perceived translation of the whole limb, possibly involving the shoulder.

Furthermore, two *explicit* measures of the perceived length of the vibrated forearm were also included: (1) ratings from a visual scale representing different arm lengths, and (2) self-reports about changes in arm sensations. Data from both explicit measures demonstrated that there was no telescoping of the arm. In the present study proprioceptive cues coming from the arm were rendered inaccurate as the perceived position of participants’ arm was not congruent with its actual position. In a previous research [9], self-localisation abilities in healthy participants were examined by comparing a condition in which visually and proprioceptively encoded positions of the hand were rendered incongruent [17], and control conditions in which they were congruent. Participants were blinded to the conditions and, even though they reported that they could not distinguish between the congruent and incongruent conditions, their self-localisation behaviour was different between conditions. This interesting aspect will need to be further investigated in future research.

As far as the self-localisation abilities during tendon stimulation are concerned, data from both Experiment 1 and Experiment 2 showed a drift towards the body in perceived fingertip location during dual tendon vibration. This aspect of our finding corroborates previous reports [7] [2, 10]. However, by measuring the arm perceived position on both x- and y-axes at the same time, we were able to extend previous results, showing that the biceps and triceps stimulation follows the arc of hand motion. In regards to the dual tendon vibration, data from Experiment 1 showed that the upward-downward axis (y-axis) translation of perceived arm location is clearly reduced, and it was the most accurate of all conditions. This has been reported in previous studies [4, 7], but raises the new possibility that localisation accuracy is enhanced when there are inconsistent proprioceptive cues (i.e. conflict between opposing directional biases). In fact, that vibration improves proprioceptive acuity has been shown for both neck and back proprioception [18]. Furthermore, our results clearly support the idea that, when no movement is performed or, as in this case, is *perceived* to be performed, a certain position is stored in memory [6]. During true movement, sensory and motor systems constantly send positional information to the brain. Thus, since no updating is required, it seems plausible that the reported position of the limb in case of dual vibration is the one stored in memory. This would suggest, then, that vibrating two antagonistic tendons and not performing any vibration at all should give rise to the same localisation response. Experiment 1, instead, showed that participants’ localisations on the y-axis were more accurate in the Dual vibration condition than they were in the No vibration condition. Under normal conditions (e.g. when vibration is not delivered) gravity would normally cause a resting forearm to extend downwards. In Experiment 1, participants’ arm was not completely at rest because it was suspended in the sagittal plane by means of a piece of fabric pinned at the panel, with the simple aim of keeping the right index finger in place (similar to Longo et al. [7]). We suspect that the participants probably had some level of biceps activity in order to avoid an excessive burden on the piece of fabric, although neither we, nor Longo et al. [7] verified this. This may have been a problem because it has been shown that sustained flexion of the biceps muscle produces changes in perceived limb position in the opposite direction (i.e. downward errors) [19, 20]. However, this possibility actually adds weight to our finding because localisation was more accurate during the dual stimulation condition than when it was simply resting–perhaps the dual vibration overrides the sustained flexor activity effect. Unfortunately, this effect cannot be fully compared with the localisation errors from Experiment 2, as in this second experiment the setup was slightly different (e.g. elbow was fully supported, hand was in a different position).

According to our results, when the biceps and triceps tendons are vibrated, not only a displacement illusion of elbow flexion and extension occur, but there is also a perceived shift of the elbow itself towards or away from the body, likely driven by an illusion of displacement involving the shoulder. This may appear in contrast with the conclusion that the effects of dual tendon vibration cannot be explained with a perceived movement backwards of the arm at the shoulder joint [7]. Nonetheless, in that experiment, participants were asked to match with the entire arm (including angles of the shoulder, elbow and wrist, see [7] Supplementary Material) the posture of the vibrated arm, but the accuracy in matching a shoulder or elbow angle is usually quite poor compared to finger localisation [21].

The present research still has some limitations. For example, we initially considered using a tactile paradigm similar to the one used by de Vignemont and colleagues [3] in order to assess the changes in perceived length of the participants’ finger. Data from pilot studies showed however, that due to very poor tactile acuity on the forearm [22], we doubted our ability to detect an effect should one be present. In addition to this, the vibration frequency that we chose may have induced an illusion of displacement but not necessarily an illusion of movement, according to the participants’ self-report (i.e. not all of them described a sensation of movement during the tendon vibration, while all of them misplaced their arm when asked to localise it). It might be that the illusion of movement is crucial to induce a telescoping effect. However, that we replicated previous results suggests against this possibility. Moreover, that the vibratory devices were handheld might have biased the nature of the illusion. Pilot trials suggested no perceived change in the vibration parameters as reported by participants. Also, even though in Longo and colleagues [7] the vibratory device was handheld as well, the adopted device was different. However, results from Experiment 1 suggest that the effect was comparable.

Finally, the nature of the localisation task should be taken into account and the use of the contralateral arm to perform the task migh constitute a possible confounder in the reported sensations and in the ability to localise participants’ own arm [23–25]. Further research will be needed to clarify these points.

In conclusion, the present study does not support the idea of telescoping limb induced by opposing proprioceptive cues, in this case during dual tendon stimulation. Instead, we argue that a general illusion of displacement of the whole arm–also at the shoulder joint level–occurs under these conditions of stimulus presentation.

## Supporting Information

S1 Appendix The S1 appendix contains Table A and B on participants’ self-reports.
(DOCX)

S1 File The Excel file named ‘Bellan et al._data’ contains 5 sheets: ‘Exp. 1’ sheet: average localisation errors (cm) for each participant (n = 13) and for condition (or vibration site, i.e. triceps, biceps, dual, none). ‘Exp. 2_localisation task’ sheet: average localisation errors (cm) for each participant (n = 15), for condition (or vibration site, i.e. triceps, biceps, dual, none) and for target (i.e. fingertip, wrist, elbow). ‘Exp. 2_perceived arm length’ sheet: average perceived arm length (cm) for each participant (n = 15), for condition (i.e. real, triceps, biceps, dual, none) and for arm segment (i.e. hand, forearm). ‘Exp.2 _visual task’ sheet: ratings for the visual scale for each participant (n = 15) and for condition (or vibration site, i.e. triceps, biceps, dual, none). ‘Exp.2 _self-report’ sheet: self-report for each participant (n = 15) and for condition (or vibration site, i.e. triceps, biceps, dual, none), both about the illusion of movement (‘Movement’) and the illusion of change in shape or length (‘Shape or length’) of the vibrated arm.
(XLSX)
