# Supplementary material for: No Telescoping Effect with Dual Tendon Vibration
Source: PLoS One. 2016 Jun 15;11(6):e0157351. doi: 10.1371/journal.pone.0157351 (PMC4909295; doi:10.1371/journal.pone.0157351)
Supplement: S1 Appendix — The S1 appendix contains Table A and B on participants’ self-reports. (DOCX) [file pone.0157351.s001.docx]

# S1 Appendix

|  | **Movement** | | | | **Shape or length** | | | |
| --- | --- | --- | --- | --- | --- | --- | --- | --- |
|  | **Triceps** | **Biceps** | **Dual** | **None** | **Triceps** | **Biceps** | **Dual** | **None** |
| **1** | up | down | down | nothing | nothing | shorter | nothing | nothing |
| **2** | up | down | nothing | nothing | nothing | nothing | fingers longer | nothing |
| **3** | nothing | nothing | nothing | nothing | nothing | strange | nothing | nothing |
| **4** | up | down | away | nothing | shorter | longer | nothing | nothing |
| **5** | nothing | away | nothing | nothing | nothing | nothing | fatter and shorter | nothing |
| **6** | nothing | away | nothing | nothing | nothing | longer | longer | nothing |
| **7** | nothing | away | towards body | nothing | nothing | longer | shorter | nothing |
| **8** | up | glass moving away | glass moving away | nothing | shorter | shorter | shorter | nothing |
| **9** | nothing | nothing | nothing | nothing | curved | curved | nothing | nothing |
| **10** | forward | forward | forward | nothing | longer | longer | curled up | nothing |
| **11** | up and down | up and down | nothing | nothing | shorter | longer | nothing | nothing |
| **12** | down | forward | side (right) | nothing | nothing | longer | nothing | nothing |
| **13** | up | down | down | nothing | nothing | nothing | narrower | nothing |
| **14** | nothing | waving | nothing | nothing | nothing | longer | nothing | nothing |
| **15** | side (left) | side (right) | nothing | nothing | nothing | nothing | nothing | nothing |

**Table A. Participants’ self-report**

|  | **Triceps** | hand | forearm | visual | **Biceps** | hand | forearm | visual | **Dual** | hand | forearm | visual |
| --- | --- | --- | --- | --- | --- | --- | --- | --- | --- | --- | --- | --- |
| **1** | nothing | -3.9 | -4.9 | 2 | shorter | 0.2 | 7.0 | -3 | nothing | 0.9 | -6.0 | -6 |
| **2** | nothing | 0.8 | -1.4 | 0 | nothing | 0.5 | 6.0 | -2 | fingers longer | 2.8 | -1.6 | -3 |
| **3** | nothing | -3.0 | -5.4 | 0 | strange | -2.2 | -4.4 | -2 | nothing | -4.9 | -2.2 | -3 |
| **4** | shorter | -6.1 | 2.3 | -3 | longer | -5.7 | 5.8 | -1 | nothing | -2.9 | 4.4 | -3 |
| **5** | nothing | -5.6 | -0.4 | 0 | nothing | -7.0 | -6.0 | -1 | shorter | -5.7 | -1.9 | -2 |
| **6** | nothing | -0.1 | 0.0 | -1 | longer | -0.7 | -1.3 | -1 | longer | -0.5 | -3.5 | -1 |
| **7** | nothing | -7.7 | -0.5 | -4 | longer | -5.1 | 0.1 | 1 | shorter | -7.9 | -3.8 | -1 |
| **8** | shorter | -5.9 | -8.3 | -4 | shorter | -5.6 | -5.2 | 1 | shorter | -5.6 | -7.1 | 0 |
| **9** | curved | -5.9 | -5.4 | 1 | curved | -6.3 | -4.3 | 1 | nothing | -5.4 | -4.0 | 1 |
| **10** | longer | -5.4 | -4.4 | 2 | longer | -4.4 | -3.1 | 1 | curled up | -3.7 | -7.2 | 1 |
| **11** | shorter | -8.4 | 2.8 | -1 | longer | -5.3 | 2.6 | 1 | nothing | -6.2 | 3.2 | 1 |
| **12** | nothing | -6.0 | -2.9 | 2 | longer | -4.8 | 2.2 | 2 | nothing | -5.5 | 3.2 | 2 |
| **13** | nothing | -4.9 | -0.9 | 1 | nothing | -4.8 | -0.9 | 3 | narrower | -1.5 | -3.5 | 2 |
| **14** | nothing | -2.4 | -4.8 | 2 | longer | 0.0 | -3.3 | 4 | nothing | -2.1 | -3.0 | 2 |
| **15** | nothing | -4.0 | -1.2 | 1 | nothing | -4.6 | 1.2 | 4 | nothing | -4.8 | -1.3 | 2 |

## Table B. Participants’ self-report on changes in shape or length of the vibrated arm compared with perceived length of hand and forearm and responses at the visual scale.
